# Novel association of APC with intermediate filaments identified using a new versatile APC antibody

**DOI:** 10.1186/1471-2121-10-75

**Published:** 2009-10-21

**Authors:** Yang Wang, Yoshiaki Azuma, David B Friedman, Robert J Coffey, Kristi L Neufeld

**Affiliations:** 1 Department of Molecular Biosciences, University of Kansas, Lawrence, KS, USA; 2 Mass Spectrometry Research Center, Vanderbilt University Medical Center, Nashville, TN, USA; 3 Departments of Cell and Developmental Biology and Medicine, Vanderbilt University Medical Center, Nashville, TN, USA

## Abstract

**Background:**

As a key player in suppression of colon tumorigenesis, Adenomatous Polyposis Coli (APC) has been widely studied to determine its cellular functions. However, inconsistencies of commercially available APC antibodies have limited the exploration of APC function. APC is implicated in spindle formation by direct interactions with tubulin and microtubule-binding protein EB1. APC also interacts with the actin cytoskeleton to regulate cell polarity. Until now, interaction of APC with the third cytoskeletal element, intermediate filaments, has remained unexamined.

**Results:**

We generated an APC antibody (APC-M2 pAb) raised against the 15 amino acid repeat region, and verified its reliability in applications including immunoprecipitation, immunoblotting, and immunofluorescence in cultured cells and tissue. Utilizing this APC-M2 pAb, we immunoprecipitated endogenous APC and its binding proteins from colon epithelial cells expressing wild-type APC. Using Liquid Chromatography Tandem Mass Spectrometry (LC-MS/MS), we identified 42 proteins in complex with APC, including β-catenin and intermediate filament (IF) proteins lamin B1 and keratin 81. Association of lamin B1 with APC in cultured cells and human colonic tissue was verified by co-immunoprecipitation and colocalization. APC also colocalized with keratins and remained associated with IF proteins throughout a sequential extraction procedure.

**Conclusion:**

We introduce a versatile APC antibody that is useful for cell/tissue immunostaining, immunoblotting and immunoprecipitation. We also present evidence for interactions between APC and IFs, independent of actin filaments and microtubules. Our results suggest that APC associates with all three major components of the cytoskeleton, thus expanding potential roles for APC in the regulation of cytoskeletal integrity.

## Background

Mutation of Adenomatous Polyposis Coli *(APC*) is an early event in colorectal carcinogenesis. The various subcellular localizations and binding partners of APC implicate this tumor suppressor in a wide variety of cellular functions in normal cells. The most well characterized function of APC is to inhibit Wnt-β-catenin signaling by forming a multi-protein complex which targets cytoplasmic β-catenin for destruction [1-3]. A role for APC in the regulation of cytoskeletal integrity has also been proposed.

APC linkage with the actin network was demonstrated by both direct interaction of APC with actin and by actin-dependent membrane-localization of APC [4,5]. Ectopic expression of APC, truncation of APC by mutation, and APC loss all result in aberrant cell migration [6-8]. Identification of APC in a complex with IQGAP, a scaffolding protein that binds to growing microtubules and regulates actin filament elongation, provides evidence that APC participates in cell migration [9,10]. Depletion of either APC or IQGAP1 inhibits actin polymerization and cell polarization [9]. APC also positively affects ASEF, a guanine nucleotide exchange factor specific for Cdc42 [11-13]. A truncated APC protein similar to those associated with colon cancers was unable to activate ASEF [12]. APC interaction with the microtubule cytoskeleton has also been established. Early localization studies identified APC at the plus ends of microtubules, implicating APC in cell migration [14,15]. The functional interaction of APC with the microtubule network is strengthened by the finding that APC directly interacts with tubulin and the microtubule-binding protein, EB1 [16-18]. Depletion of APC destabilizes microtubules and inhibits spindle formation and cellular protrusions, thereby compromising cellular migration [19].

Actin-containing microfilaments, microtubules, and intermediate filaments (IFs), constitute the three main cytoskeletal elements that act coordinately to regulate cell structure, polarity and migration. IFs function as a scaffold to maintain cell and tissue integrity and defects in IF impact a number of diseases (see review [20]). In the present study, we provide further evidence for the involvement of APC in the regulation of cytoskeletal structure through interaction with IFs. Using purified polyclonal sera raised against the 15 amino acid repeat region of APC (referred to APC-M2 pAb), we identified interactions between APC and IF proteins lamin B1, lamin B2, keratin 81 and keratin 82. We verified the lamin B1 interaction with APC by co-immunoprecipitation as well as by immunofluorescence microscopy in both cultured cells and human colonic tissue. Nuclear lamins are type V IFs that form a spherical mesh lining the nuclear envelope, providing attachment sites for chromosomes and nuclear pores [21]. The keratin/APC interaction was further supported by protein co-localization in cultured cells. Keratins are type I and II IFs that are predominantly found in epithelial cells, providing structural integrity to those cells so that they can withstand mechanical stress [22]. Identifying an interaction between APC and IFs expands the potential role of APC in cytoskeletal regulation. Although APC expression and functions have been widely studied in basic research and clinical settings, recent reports have raised questions about the accuracy and reliability of many commercially available antibodies [23,24]. Therefore, an APC antibody that accurately determines levels and subcellular localizations of APC in cells and especially in tissues is extremely important. Our characterization of the APC-M2 pAb validates its use in a variety of applications including detection of APC protein in mouse and human tissues.

## Results

### Intermediate Filament protein lamin B1 co-precipitates with APC using the new APC-M2 pAb

**Figure 1:**
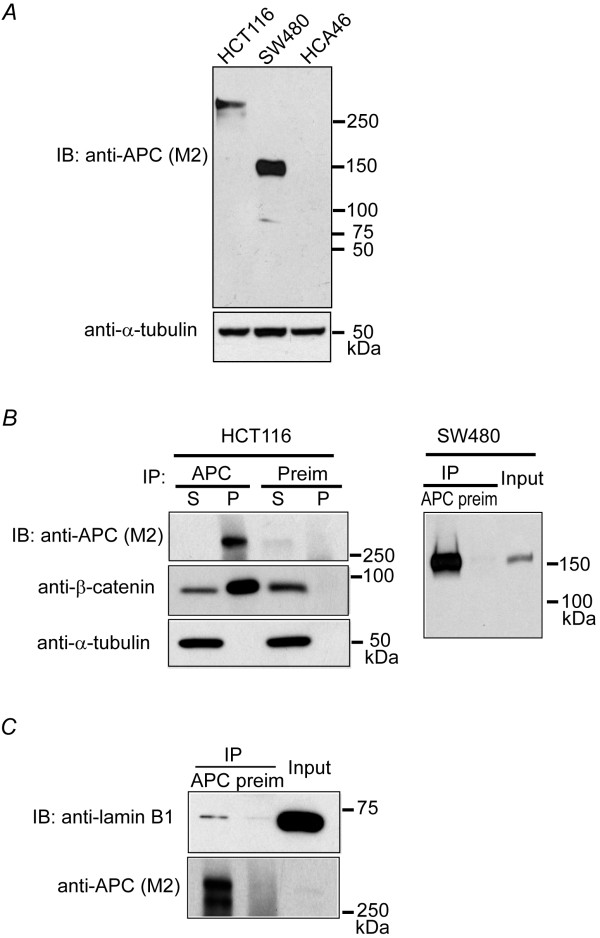
**Lamin B1 co-precipitates with endogenous APC using APC-M2 pAb**. (A) APC-M2 pAb specifically detects full-length (~310 kDa) APC in HCT116βw cell lysates and truncated (~150 kDa) APC in SW480 cell lysates by immunoblot. APC-M2 pAb does not detect APC in HCA46 cell lysates, null for APC. Equivalent total protein (30 μg) was resolved in each lane. Blot shows entire spectrum of proteins from > 300 kDa to ~15 kDa. α-tubulin is shown as a loading control. (B) APC-M2 pAb immunoprecipitates APC from HCT116βw (left panel) and SW480 (right panel) cell lysates. β-catenin co-precipitates with full-length APC (left panel, middle blot). Neither APC nor β-catenin co-precipitated with preimmunue sera (Preim). P, precipitated proteins from 270 μg protein lysates; S, 10% of non-precipitated supernatant proteins (27 μg). Input, 5% of total input proteins (25 μg). (C) Endogenous lamin B1 co-precipitated with APC using APC-M2 pAb, but not preimmune sera. Precipitated proteins from 376 μg protein lysates were loaded. Input, 6% of total input proteins (37.5 μg). The lower band in the APC IP lane represents degradation products.

As a multi-functional tumor suppressor protein, APC has been widely analyzed regarding its subcellular localization and its interaction with other proteins. Most commercial antibodies, which were raised against either the N- or C-terminus of APC, recognize proteins other than APC [23,25]. This cross reactivity makes it difficult to obtain reliable data using standard molecular and cellular biology techniques to study APC. Thus, we injected rabbits with purified protein corresponding to the central domain of APC (amino acids 959-1338, Additional File 1) and we affinity-purified sera from these rabbits (referred to hereafter as APC-M2 pAb). Our initial analysis of APC-M2 pAb in cultured cells utilized HCT116βw colon epithelial cells that possess only the wild-type β-catenin allele and thus differ from the parental HCT116 cells that contain one wild-type and one mutant β-catenin allele [26]. APC-M2 pAb recognizes full-length APC which appears as a single band of ~310 kDa in lysates from HCT116βw cells and truncated mutant APC (~150 kDa) in lysates from SW480 cells (Figure 1A). No band was detected in lysates from HCA46 cells which are essentially null for APC [27]. Both full-length and truncated APC were efficiently precipitated using APC-M2 pAb (Figure 1B). A well established binding partner of APC, β-catenin, was also co-precipitated with full-length APC from HCT116βw cell lysates (Figure 1B). Thus, we confirmed the reliability of APC-M2 pAb for use in immunoblotting and immunoprecipitation.

APC is a multi-functional protein involved in several different cellular pathways. Many of APC's functions are only beginning to be elucidated. Identifying novel binding partners for APC is a step toward fully understanding APC's involvement in the maintenance of cellular integrity. Therefore, APC-M2 pAb was used to co-immunoprecipitate APC and its associated proteins from HCT116βw cell lysates. Nine protein bands that were unique to the APC co-precipitation and not observed using preimmune sera as a control were visualized by colloidal blue staining (Additional file 2). These bands and corresponding regions from the control lane were separately isolated, pooled and analyzed by Liquid Chromatography Tandem Mass Spectrometry (LC-MS/MS). Forty-three potential APC binding proteins that precipitated with APC antisera and not with preimmune sera were identified as having cross-correlation scores (x-corr) of ≧2 for 2+ ion and ≧2.5 for 3+ ion (Additional file 3). These 43 proteins could be grouped in eight broad categories, the largest two being cytoskeletal regulation and RNA processing/translation. From the most abundant potential APC-binding proteins found, we chose to examine the interaction of APC with intermediate filaments in more detail. IF proteins identified were lamin B1, lamin B2, keratin 81 and keratin 82 (Table 1). Initially, we focused on the most abundantly APC-associated protein of the four, lamin B1. Interaction of lamin B1 with APC was verified by co-immunoprecipitation (Figure 1C).

**Table 1:** Intermediate filament proteins associated with APC as identified using LC-MS/MS

**Protein**	**Accession No**	**Peptides found 1^st^/2^nd ^run**
Lamin B1	Q6DC98	7/6
Lamin B2	Q03252	2/3
Keratin 81	Q14533	5/5
Keratin 82	Q9NSB4	2/2

(at least 2 peptides found in each duplicate run; all peptides passed Xcorr cutoffs of 2 for +2 ion and 2.5 for +3 ion)

### Lamin B1 and keratin colocalization with APC revealed using APC-M2 pAb

**Figure 2:**
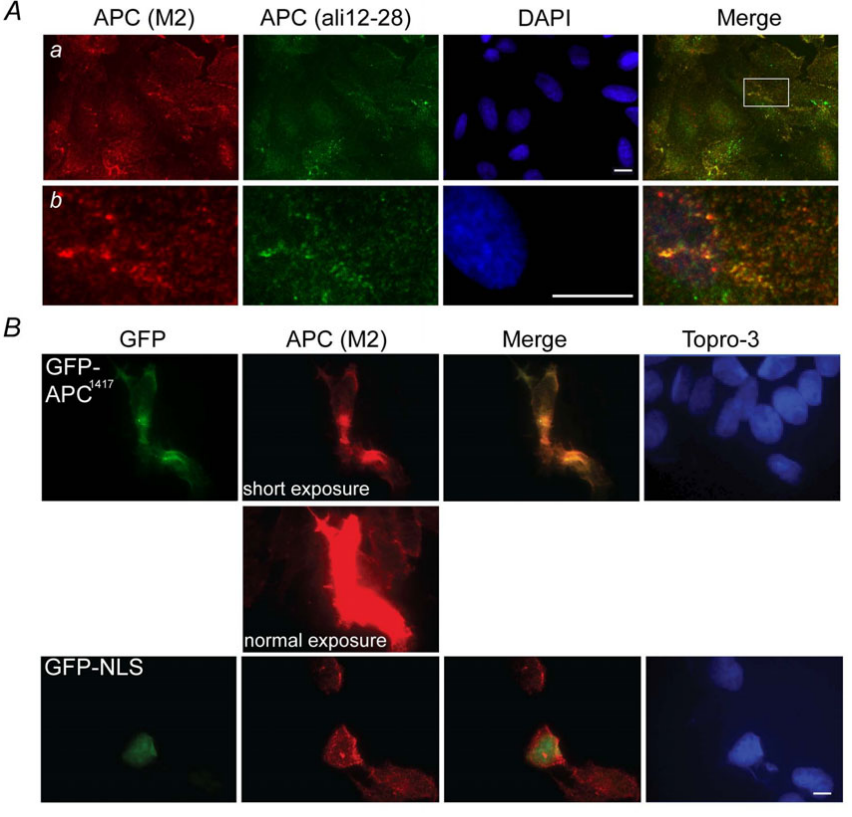
**APC-M2 pAb recognizes endogenous and exogenous APC protein by immunofluorescence microscopy**. (A) U2OS cells double-labeled with APC-M2 pAb (red) and commercial anti-APC (ali12-28) mAb (green). (b) Magnified image of a region indicated in (a) shows considerable overlap of red and green signals. Scale bars, 5 μm. (B) Colocalization of APC^1417 ^fused-GFP (green, upper panel) or NLS (nuclear localization signal from SV40) fused-GFP tag (green, lower panel) with APC-M2 pAb (red) signal. Short exposure revealed APC-M2 pAb signal overlapping that of GFP-APC^1417^. Longer (normal) exposure led to saturated signal in GFP-APC^1417^-expressing cells but allowed visualization of endogenous APC staining with APC-M2 pAb in adjacent non-transfected HCT116βw cells. Note that there is no overlap of APC-M2 pAb labeling with NLS fused GFP signal. Scale bar, 5 μm.

To validate the interaction of lamin B1 with APC using colocalization, we first verified the utility of APC-M2 pAb for immunofluorescence microscopy. APC-M2 pAb recognizes APC at cell-cell junctions, in the nucleus and in the cytoplasm of U2OS cells (Figure 2A). This pattern showed considerable overlap with that of one of the most frequently utilized commercially available APC antibodies, ali12-28, which was raised against amino acid 135-422. Moreover, APC-M2 pAb effectively detected a truncated APC protein expressed as a GFP-fusion in HCT116βw cells (Figure 2B). Because the exogenous GFP-fused APC was greatly over-expressed, the exposure time for the APC-M2 pAb signal was shortened to reveal the overlapping pattern. In contrast, APC-M2 pAb did not recognize GFP fused to the SV40 Nuclear Localization Signal (NLS).

**Figure 3:**
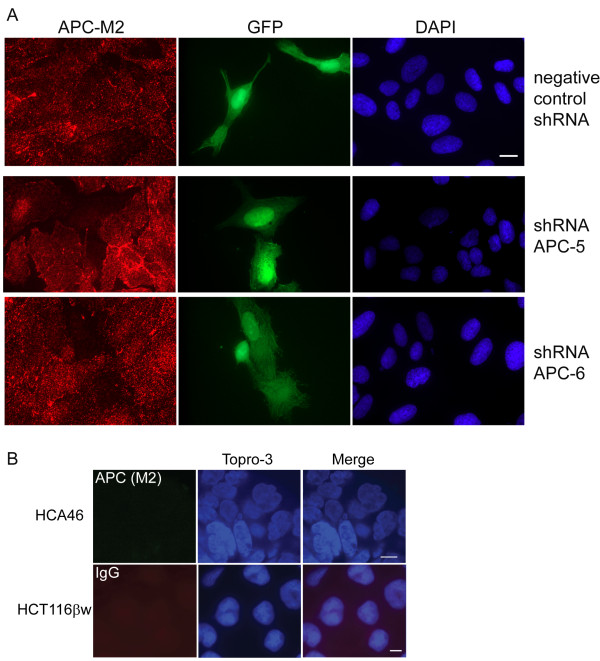
**APC-M2 pAb immunofluorescence signal reduced by APC-shRNA**. (A) U2OS cells transfected for 48 hours with negative control shRNA or one of two individual shRNA plasmids specific for *APC *were probed using APC-M2 pAb. GFP (green) expression from the shRNA vector marks transfected cells. Expression of either shRNA-APC number 5 or 6 resulted in reduction of the majority of the nuclear and cytoplasmic APC-M2 pAb signal. Scale bar, 5 μm. (B) Immunofluorescence microscopy using APC-M2 pAb to probe HCA46 cells, null for APC (upper panel), or purified rabbit IgG to probe HCT116βw cells (lower panel) reveals only minimal signal. Scale bars, 5 μm.

To further validate the specificity of APC-M2 pAb, U2OS cells were transfected with a plasmid containing GFP and either a small hairpin RNA (shRNA) specific for *APC *or a negative control shRNA. Thus, cells expressing the shRNA plasmids can be identified by the presence of GFP. Cells expressing the negative control shRNA plasmid displayed no alteration in APC signal intensity using APC-M2-pAb for immunofluorescence microscopy (Figure 3A). In contrast, cells expressing either of two *APC*-specific shRNA plasmids displayed a marked reduction in both cytoplasmic and nuclear APC signal intensity (Figure 3A). As another critical negative control, APC-M2 pAb did not reveal immunofluorescence signal above background in HCA46 cells which do not express APC (Figure 3B). The slight fluorescent signal seen in HCA46 cells stained using APC-M2 pAb or in HCT116βw cells stained using purified rabbit IgG (Figure 3B) was similar to that of control cells stained with only secondary antibody (Additional file 4).

**Figure 4:**
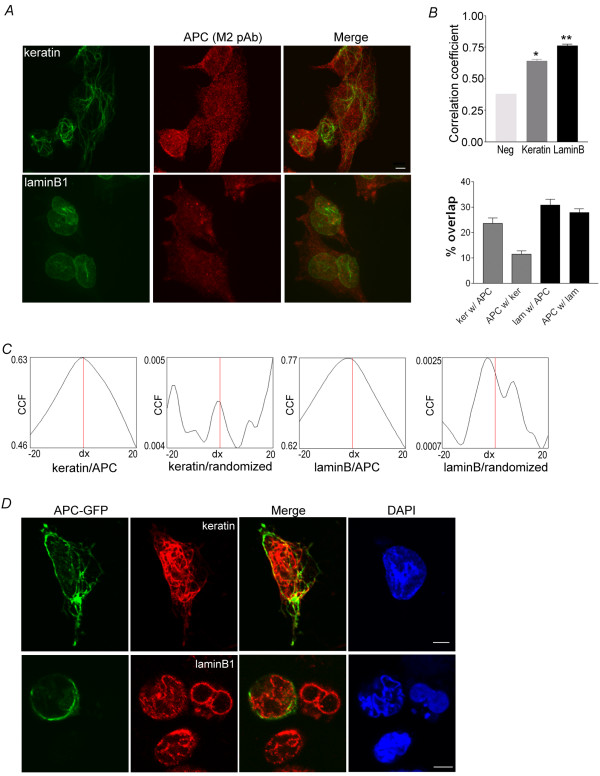
**IF proteins lamin B1 and keratins colocalize with endogenous and exogenous APC in cultured cells**. (A) Confocal immunofluorescence microscopy reveals partial overlap of APC (red) and keratin (green) or lamin B1 (green) in HCT116βw cells. Single projections of 20 images resolved along the z-axes for lamin and 30 images for keratin are shown. Scale bar, 4.6 μm. (B) More than 700 individual images were analyzed for each protein pair examined to determine the Pearson's correlation coefficient (upper graph) or the Manders overlap coefficient (lower graph). Additional file 4B shows representative image of the negative control (both secondary antibodies with no primary antibody). The average Pearson's correlation coefficient ± SEM calculated from n individual z-series images (n = 900, keratin/APC; n = 720, lamin B1/APC) were significantly higher than the correlation coefficient determined for the negative control (* and ** p < 0.0001). (C) The Van Steeples cross correlation function (CCF) was plotted as a function of image offset in pixels. Keratin and lamin B1 each show a peak of correlation with the APC signal in the original position with no offset (dx, red line). In contrast, the CCF is low, with multiple peaks for keratin or lamin B1 with a randomized APC signal. (D) HCT116βw cells expressing full-length APC fused to GFP were stained for keratin or lamin B1 (red). Confocal images show partial overlap of the keratin and lamin B1 signals with GFP. Scale bars, 5 μm.

Having validated APC-M2 pAb for use in immunofluorescence applications, we probed HCT116βw cells for endogenous APC and IF proteins lamin B1 and keratin. In single confocal slices, APC appeared in puncta that partially overlapped lamin B1 and aligned with some keratin filaments (data not shown). Figure 4A shows APC and either keratin or laminB1 signals each captured in 20 z-plane images and projected into a single image. Because the APC signal did not completely coincide with that of keratin or laminB1, quantitation of the overlap was performed by determining the Pearson's correlation coefficient from ~700 individual confocal images representing >65 individual cells captured in three-dimensions (Figure 4B). Correlation coefficients of APC/lamin B1 and APC/keratin pairs were significantly higher than that of negative control samples stained with only the secondary antibodies (Figure 4B and Additional file 4C). On average, 11% of the APC signal overlapped that of keratin and 27% overlapped that of lamin B1 (Figure 4B). In Figure 4C, images were analyzed for random overlap by shifting the green and red signals by 20 pixels in the x-direction. Offset in either direction resulted in a decrease in the correlation, indicating that the overlap is not a chance occurrence from two relatively abundant signals (Figure 4C, 1^st ^and 3^rd ^panels). In addition, the cross-correlation coefficient for keratin or lamin B with a randomized APC signal was low and showed multiple peaks (Figure 4C, 2^nd ^and 4^th ^panels). Results from the image analysis validated the observed co-localization of APC with both lamin B and keratin. As an alternative approach to further investigate this co-localization, full length APC was expressed as a GFP fusion. In single confocal slices, the GFP signal displayed partial overlap with both keratin and lamin B1 (Figure 4D). Taken together, the observed cellular co-localization is consistent with an association between APC and IF proteins keratin and lamin B1.

### Lamin B1 colocalization with APC in human colonic tissue revealed using APC-M2 pAb

**Figure 5:**
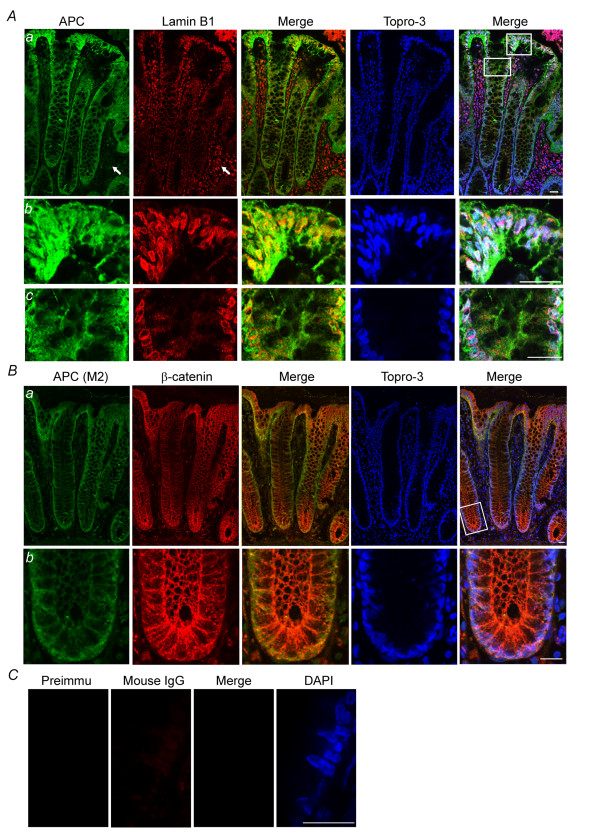
**APC colocalizes with lamin B1 in human colonic tissue**. (A) Confocal immunofluorescence microscopy of cryosections from normal human colon tissues triple-labeled with APC-M2 pAb (green), anti-lamin B1 (red) and DNA dye topro-3 (blue). Arrows point to stromal cells in which lamin B1 staining is more intense compared to adjacent epithelial cells within crypts. In contrast, less APC staining is detected in stromal cells compared to crypt epithelial cells. Tissues are oriented such that luminal surfaces are at the top of the images. (b) and (c) Enlarged images of two regions indicated in (a). (B) APC colocalizes with β-catenin in the cytoplasm and cell junctions of human colonic crypts. (b) Enlarged image of a region at the base a colonic crypt shown in (a). (C) Immunofluorescence microscopy using preimmune sera and purified mouse IgG to probe consecutive tissue sections revealed only minimal signal. Scale bars, 20 μm.

In normal human colon tissue, APC-M2 pAb predominantly marked the cell junctions, with increasing cytoplasmic and nuclear expression in epithelial cells at the luminal surface (Figure 5A-a). Lamin B1 showed perinuclear expression in epithelial cells in the upper half of the crypts and also in mesenchymal cells located between crypts. Both APC and lamin B1 exhibited increasing expression from the crypt base toward the luminal surface (Figure 5A-a). A higher magnification of the confocal image reveals perinuclear colocalization of APC with lamin B1 at the luminal surface (Figure 5A-b). A similar co-localization could be seen further down the crypt where the APC signal was not as saturated (Figure 5A-c). Unlike lamin B1 staining, APC staining appeared more intense in epithelial cells than in surrounding stromal cells. As a positive control, normal human colonic tissue was co-stained for β-catenin and APC (Figure 5B). APC-M2 pAb revealed APC in both nuclei and at cell junctions, with a staining pattern partially overlapping that of β-catenin. Human colon tissue stained with rabbit preimmune sera and purified mouse IgG revealed only minimal signal and provided a negative control (Figure 5C).

### APC association with intermediate filament proteins is not dependent on actin or tubulin

**Figure 6:**
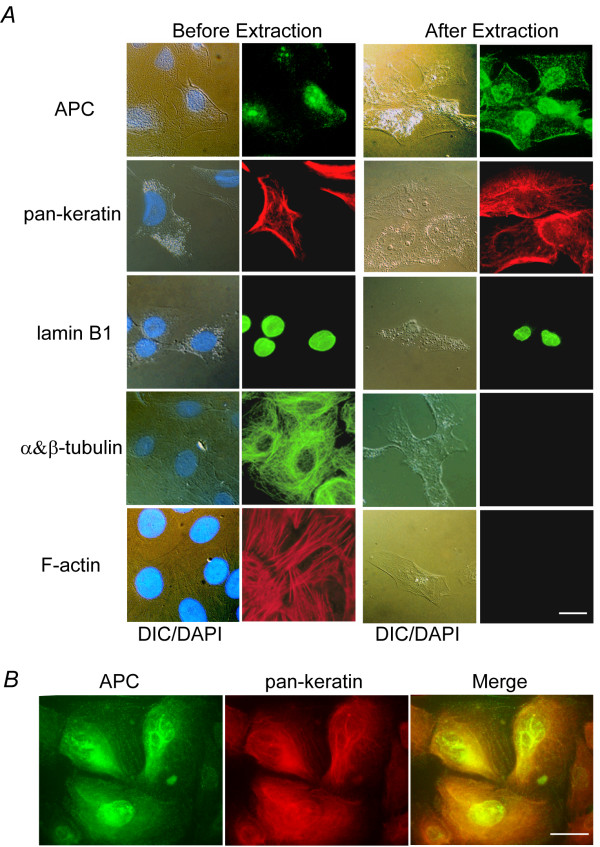
**APC remains associated with IF proteins following extraction of actin and tubulin**. (A) Left two panels, 184A1 epithelial cells analyzed by immunofluorescence microscopy reveal distribution of APC, lamin B1, keratin, tubulin, actin (detected with phalloidin) and DNA (blue in DIC overlay). Right two panels, following sequential extraction, APC remained cell associated, along with IF proteins lamin B1 and keratin. Note that actin, tubulin, and DNA were successfully extracted from these cells. Nuclei are indicated by DAPI staining. (B) Colocalization of APC (green) with keratins (red) after cell extraction. Scale bars, 10 μm.

IFs interact with both actin-containing microfilaments and microtubules (see review [28]). Because APC also associates with actin and microtubules, it is possible that the co-immunoprecipitation of IF proteins with APC is due to precipitation of a large complex of cytoskeletal components. To determine whether the APC/IF interaction was dependent on actin-containing microfilaments and microtubules, we performed a traditional solubilization and extraction of both tubulin- and actin-containing microfilament proteins from cells, leaving behind predominantly insoluble IFs and desmosomes [29]. After sequential extraction, the cytoskeletal proteins tubulin and actin were efficiently removed from cells as determined by immunofluorescence microscopy (Figure 6A). APC, however, remained cell-associated, as did IF proteins lamin B1 and keratin. Colocalization of APC with keratins was apparent in extracted cells, with the APC appearing as puncta along the keratin filaments (Figure 6B). That APC remains cell-associated following this extraction procedure suggests a stable interaction between APC and IFs, independent of actin-containing microfilaments and microtubules.

### APC-M2 pAb is a versatile antibody, specific for APC

**Figure 7:**
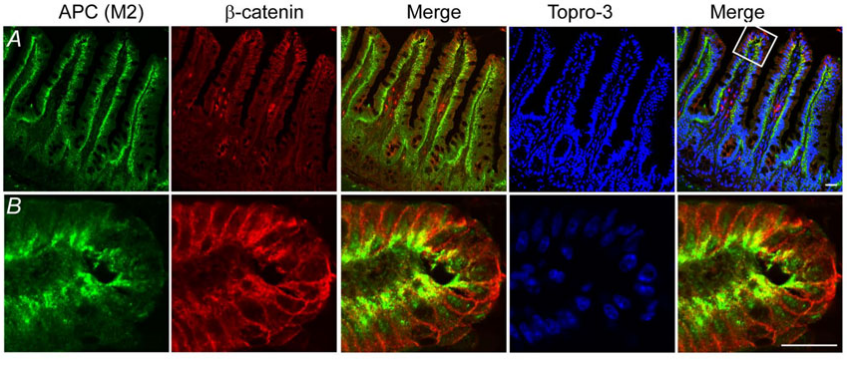
**APC-M2 pAb recognizes endogenous APC protein in normal mouse intestinal tissue**. Confocal immunofluorescence microscopy of cryosections from wild-type mouse ileum triple-labeled with APC-M2 pAb (green), β-catenin mAb (red) and DNA dye topro-3 (blue). Tissues are oriented so that luminal surfaces are at the top of the images. (A) An increasing gradient of APC staining from crypts to villus tips is apparent at this magnification. (B) Enlarged image of a region at the villus tip shown in (A). APC staining is concentrated in basolateral regions of villus epithelial cells. Scale bars, 20 μm.

We have demonstrated specificity of APC-M2 pAb for APC protein by immunoblot (Figure 1A), immunoprecipitation (Figure 1B-C and Additional file 3) and immunofluorescence confocal microscopy using both cultured cells and human tissue (Figure 2, 3, and 5). Many mouse models have been established to analyze APC functions in the context of a whole mammalian organism. To validate the use of APC-M2 pAb in animal studies, frozen sections of mouse small intestinal tissue were probed with APC-M2-pAb and then APC was visualized using confocal microscopy. The staining of APC was most intense in cells at the top of villi, with less APC in cells near the crypt base (Figure 7A). APC appeared at cell-cell junctions and in the cytoplasm with occasional nuclear localization (Figure 7). In the villus, cytoplasmic puncta were also visible, particularly near the basal surface (Figure 7B). Of note, APC-M2 pAb did not reveal the apical APC distribution in intestinal tissues which was previously attributed to cross reactivity with a non-APC protein [23].

## Discussion

In the current study, we have generated purified polyclonal antiserum that is specific for APC. APC-M2 pAb is versatile and can be used to detect APC in immunostaining, immunoblotting and immunoprecipitation. Using this antibody, we identified a novel interaction between APC and IFs. The APC and IF protein interactions were confirmed by co-precipitation and colocalization using both endogenous and GFP-tagged exogenous APC in cultured epithelial cells. Association of APC with IFs is not dependent on actin-containing microfilaments and microtubules.

APC mutations are found in over 80% of all colorectal cancers and have been assessed for screening and prognosis purposes in the clinical setting. For such research, access to an APC antibody that accurately depicts levels and subcellular localizations of APC in cells and especially in tissues is extremely important. According to Wakeman *et. al. *[23], many commercially available antibodies also recognize an unidentified protein that localizes to the apical region of polarized epithelial cells. Most currently available commercial APC antibodies were raised against several hundred amino acids at either the N- or C-terminus of the APC protein. However, most of these antibodies have not been proven to be specific for APC [23]. Furthermore, many of these antibodies are only useful for a single application. For example, anti-APC Ab-7 is only effective when used for immunostaining. Therefore, we generated a new antibody against the 15 amino acid repeat region in the middle of APC (Additional file 1). The affinity-purified APC-M2 pAb recognizes junctional, cytoplasmic and nuclear APC by immunostaining (Figure 2A) without producing the non-specific apical staining pattern previously seen [23]. Although there was a great deal of overlap, immunofluorescent signals from APC-M2 pAb and anti-APC ali12-28 were not 100% coincident. The ali12-28 antibody was raised against a different portion of APC protein than M2-APC. The most likely explanation for the slight signal disparity is that the epitopes recognized by APC-M2 pAb are masked by APC folding, or the binding of another protein to a subset of the APC protein. In a similar manner, it would be expected that the epitope recognized by ali12-28 might be inaccessible in some fraction of the APC protein.

One of the most clinically relevant applications for APC antibodies is for immunohistochemical analysis of tissues. We have validated the use of APC-M2 pAb for immunostaining sections of frozen human colonic tissue and mouse small intestinal tissues. Previously, APC has been detected in both the nucleus and cytoplasm of human colonic tissue, with more prevalent cytoplasmic staining in colonic tumors compared to normal tissue [30]. In our study of human colon tissue, APC-M2 pAb revealed APC in both nuclei and at cell-cell junctions, partially colocalized with β-catenin, similar to previous reports [14,30] (Figure 5B). APC protein also appeared concentrated near the basal surface of epithelial cells (Figure 5A and 5B) as previously reported [31]. APC staining became most intense in cells at the top of villi in the mouse small intestine (Figure 7A-B) and in cells at the luminal surface of the human colon (Figure 5A), consistent with a role for APC in differentiated cells. Most importantly, APC-M2 pAb did not recognize protein at the apical surface of intestinal tissues. Thus, APC-M2 pAb does not appear to exhibit the cross reactivity previously reported for some commercially available APC antibodies [23]. Taken together, the APC-M2 pAb is a useful antibody for future examination of APC in mouse and human tissue sections. At present, APC-M2 pAb does not appear to recognize APC in formalin-fixed paraffin-embedded tissues (data not shown).

Intermediate filaments are essential components of cytoskeletal structure. Recent reports have implicated APC in the regulation of cellular structural integrity through affects on microtubule growth directionality and polarity [32,33]. APC also associates with microtubule-interacting protein EB1, and actin-associated protein ASEF [11,18]. Our own previous screen for APC-interacting proteins identified keratin 18 (Stecklein and Neufeld, personal communication). Although classified as a hair cuticle-specific keratin, keratin 81 is expressed in a wide variety of tissues including mammary gland and lung and in human embryonic kidney 293T cells [34-37]. Keratin 82 is also thought to be expressed predominantly in hair and nail. However, when flag-tag specific antibodies were used to immunoprecipitate flag-tagged human homolog 3 of Drosophila Disc-large (hDlg) from human embryonic kidney 293 cells, keratin 82 was identified as a protein partner [38]. This observation is significant because it indicates that keratin 82 has a broader expression pattern than initially suggested and because hDlg protein itself is a binding partner of APC [39].

Downregulation of β-catenin activity by APC is not limited to the cytoplasmic destruction complex. Association of APC with β-catenin in the nucleus appears to result in sequestration of β-catenin from the TCF/LEF transcription factor leading to inactivation of Wnt-responsive genes [40]. Furthermore, chromatin immunoprecipitation analysis revealed that APC associated with the enhancer region of a Wnt-responsive gene in a similar temporal manner as several co-repressors, suggesting that APC can oppose a Wnt signal by associating with co-repressors at the transcription complex [41]. Given the emerging role of the nuclear lamina in gene regulation [42], it is tempting to speculate that APC association with lamin B1 is yet another mechanism by which expression of Wnt-responsive genes is regulated. Moreover, finding APC associated with the nucleus after extraction suggests APC might be bound to the nuclear matrix. This observation is consistent with our previous finding that the majority of nuclear APC purified with the nuclear matrix [43].

As a vital part of the cytoskeleton, intermediate filaments are dynamic and can stabilize cellular organelles such as the nucleus [44]. Mutations in genes encoding IF proteins are associated with a number of diseases such as Monilethrix (K81) [45], Autosomal dominant leukodystrophy (LMNB1) [46], acquired partial lipodystrophy (LMNB2) [47], and various other lipodystrophies and cardiomyopathies [20]. APC potentially cross-links IFs to improve their stability or binds IFs to other structures such as actin filaments or microtubules. Therefore, truncated APC found in the majority of colorectal cancers and also in other cancers, might have a compromised ability to link APC binding partners with different IF proteins. It is worth noting that full-length APC has been found near IFs in epithelial cells from human colon tissue using immunogold electron microscopy [48]. Given the evidence for an association of APC with IFs, it is predicted that disruption of IFs would result in APC redistribution. Our attempts to disrupt IFs by treating cells with acrylamide led to cell shrinking, but not elimination of IFs (data not shown). Further study of this novel APC/IF interaction using a related approach might illuminate the underlying mechanisms of intermediate filament protein-associated diseases.

## Conclusion

In summary, we identified an interaction between APC and intermediate filament proteins lamin B1 and keratin using a novel antibody, APC-M2 pAb. The APC-M2 pAb antibody is specific for APC, versatile, and reliable, with potential value in the clinical setting and in translational research. By sequential extraction of cytoskeletal components, we have shown that the association of APC with IF proteins is not dependent on actin and tubulin. Finding APC associated with IF proteins confirms and expands upon the previous notion that APC is in close association with the cytoskeleton, raising the possibility that APC functions in the maintenance of cytoskeletal structure and integrity.

## Methods

### Cell culture, transfection, and tissue preparation

HCT116βw (a generous gift from Bert Vogelstein) and U2OS cells (ATCC) were grown in McCoy's 5A media (Gibco) supplemented with 10% Fetal bovine serum (FBS) (Hyclone). SW480 cells (ATCC) were grown in RPMI 1640 media (Cellgro) supplemented with 10% FBS. HCA46 cells (a generous gift from Ian Tomlinson) were grown in high glucose DMEM media (Gibco) supplemented with 10% FBS. 184A1 cells (a generous gift from Martha Stampfer) are immortalized human mammary epithelial cells [49], and were grown in MCDB 170 media (Clonetics) supplemented as described [50]. Lipofectamine 2000 (Invitrogen) was used to transfect cells with various GFP fusion expression constructs and SureSilencing shRNA plasmids specific for *APC *(shRNA-5 and shRNA-6) or non-specific as a negative control (SABiosciences). Colon and ileum tissues were removed from an 8-week old male C57/BL6 mouse (Charles Rivers Laboratory) immediately after CO_2 _asphyxiation, rolled into "Swiss rolls", embedded in OCT medium (Sakura Finetek) and frozen in ethanol-dry ice. Fresh normal human colonic tissue was immediately embedded in OCT medium and frozen in ethanol-dry ice.

### Antibodies, immunostaining and confocal microscopy

Tissue cryosections or cultured cells were fixed in 4% paraformaldehyde in PBS. Immunostaining of fixed tissue cryosections and cultured cells was performed as described [43,51,52]. Briefly, post-fixation samples were incubated with blocking buffer (5% normal goat serum, 1% bovine serum albumin and 0.1% triton X-100) for one hour at room temperature. Samples were then incubated with primary antibodies diluted in the blocking buffer at 4°C overnight, followed by 3 washes in PBS for 10 minutes each. Finally, samples were stained with secondary antibodies diluted in blocking buffer for 30 minutes followed by 3 washes in PBS. To better preserve IF structures, cells were incubated at -20°C for 20 minutes in 100% EtOH following the fixation step. Coverslips were mounted on slides with Prolong Antifade (Invitrogen). Antibodies used for immunostaining include APC-M2 pAb (1;1000), anti-APC (1:20, ali12-28, Calbiochem), anti-β-catenin (1:200, Transduction Lab), anti-lamin B1 (1:50, Calbiochem), anti-pan-keratin (1:50, Biomeda), Rhodamine-phalloidin (1:500, Invitrogen), and a cocktail of mAbs against anti-α&β-tubulin (a generous gift from Dave Gard); secondary antibodies used were goat anti-mouse IgG FITC (1:200, Molecular Probes), goat anti-mouse IgG Alexa 488 (1:1000, Molecular Probes), goat anti-rabbit IgG Alexa 568 (1:500, Molecular Probes), and goat anti-mouse IgG Alexa 568 (1:500, Molecular Probes). DNA was labeled with Topro-3 (1:1000, Molecular Probes) or DAPI (1:1000, Molecular Probes). Negative controls included incubation with secondary antibody alone, or replacing primary antibodies with purified IgG/preimmune sera, or secondary antibody with primary antibody of opposing species (e.g. Alexa 488 anti-mouse IgG with APC-M2-pAb, Additional file 4). These negative controls resulted in only minimal signal.

Immunostained cells or tissues were visualized using a PlanNeofluor 40X/1.3 oil objective on a Carl Zeiss Laser Scanning Microscope (LSM) 510. Images were analyzed using Zeiss LSM Image Browser Version 4.2. Images of human colon sections were captured as 2(X) by 3(Y) sub-images and automatically reassembled as one montage image.

### Protein colocalization analysis

For three dimensional analyses of immunofluorescent signals, images were captured using a Yokugawa-type spinning disk confocal microscope equipped with an Olympus 100× objective (Olympus and Intelligent Imaging Innovations, Denver, CO). For each protein pair analyzed, >65 cells were randomly selected to be imaged. Twenty to thirty image frames were collected at z-intervals of 100 nm for each cell. Raw confocal image series were analyzed by ImageJ program (NIH, Bethesda, MD) and the JACoP plugin without further processing [53]. Colocalization coefficients were calculated using Pearson's correlation coefficient or Manders' coefficient as described [51] and were plotted as mean ± SEM using GraphPad Prism software. For calculation of Van Steensel's cross-correlation function (CCF), raw confocal images from multiple z-planes were analyzed by JACoP plugin. APC images were subjected to 200 rounds of pixel randomization to generate randomized APC images to use as negative controls for the CCF.

### Immunoprecipitation and immunoblots

Purified recombinant His-S-M2-APC was resolved on a 4-12% NUPAGE gel (Invitrogen) and stained using a Colloidal Blue Staining Kit (Invitrogen) according to manufacturer's protocol. Immunoprecipitation (IP) and immunoblots (IB) were performed using modified standard protocols. Cells at 90% confluency were lysed in buffer containing 50 mM Tris (pH 7.5), 0.1% NP-40, 100 mM NaCl, 1 mM MgCl_2_, 5 mM EDTA, protease inhibitor cocktail (Sigma) and Halt phosphatase inhibitor cocktail (PIERCE) on ice for 30 minutes. Cell lysates were sonicated for 10 pulses at level 1 with 10% output 3 times using an ultrasonic homogenizer (Branson Sonifier 450). Soluble lysates were obtained by centrifugation at 17,000 × g for 20 minutes at 4°C. Specific antibodies were first pre-incubated with Protein-A dynabeads (Invitrogen) for 2 hours at room temperature. Then antibody saturated dynabeads were washed with 0.2 M triethanolamine (pH 8.0) twice and crosslinked using 4 mM DMP (PIERCE) in 0.2 M triethanolamine (pH 8.0) for 15 minutes. Crosslinking was stopped by adding 1/10 volume of 1 M Tris (pH 8.0) and incubating for 2 hours. For each reaction, 1 ug of antibody crosslinked to dynabeads was added to 1 mg of soluble lysate and incubated overnight at 4°C. Immunoprecipitated proteins were subject to two washes of 15 minutes using lysis buffer and two washes using PBS-T at 4°C. Immunoblots were probed with: purified anti-APC-M2 (1:5000), anti-lamin B1 (Oncogene, 1:1000), anti-β-catenin (Sigma, 1:2000), and anti-α-tubulin (Oncogene, 1:2000).

### Proteomic analysis

Proteins co-precipitated with purified APC-M2 antibody or preimmune sera were resolved on a 4-12% NUPAGE gel (Invitrogen) and stained using a Colloidal Blue Staining Kit (Invitrogen) according to manufacturer's protocol. The nine gel regions from each sample indicated in "Additional file 2" were excised and combined into experimental and negative control samples respectively. These were then equilibrated in 50 mM NH_4_HCO_3_, reduced with 3 mM DTT in 100 mM NH_4_HCO_3 _at 37°C for 15 minutes, and alkylated with 6 mM iodoacetamide in 50 mM NH_4_HCO_3 _for 15 minutes. The gel pieces were then dehydrated with acetonitrile and rehydrated with 15 μl of 12.5 mM NH_4_HCO_3_containing 0.01 μg/μl Modified Trypsin Gold (Promega). Trypsin digestion was carried out for >2 hours at 37°C. Peptides were extracted with 60% acetonitrile/0.1% formic acid, dried by vacuum centrifugation and reconstituted in 30 μl of 0.1% formic acid. Each peptide hydrosylate (5 μl) was analyzed by C18 reverse-phase LC-MS/MS using a Thermo LTQ Ion Trap Mass Spectrometer equipped with a Thermo MicroAS Autosampler and Thermo Surveyor HPLC Pump, nanospray source, and Xcalibur 1.4 instrument control essentially as described [54]. Tandem MS data were collected from independent duplicate experiments, and analyzed with the Sequest algorithm to search the unihuman2 database (Jan 23 2007, 223514 entries) using Xcorr cutoffs of ≧2 for [M+2H]2+/2 ions and ≧2.5 for [M+3H]3+/3 ions [55]. In addition, the database contained a concatenated reverse decoy database to estimate false-discovery rates, which were 5% or below. Proteins identified from negative control samples were subtracted from the experimental samples. Only proteins that appeared in both duplicate experiments were displayed in Table 1 and Additional file 3.

### Sequential extraction of cytoskeletal elements

The protocol for sequential extraction of cells was adapted from protocols previously described [29]. Briefly, cells were sequentially incubated with cytoskeletal buffer [10 mM PIPES pH 6.8, 300 mM sucrose, 100 mM NaCl, 3 mM MgCl_2_, 1 mM EGTA and 0.5% Triton X-100] and extraction buffer [10 mM PIPES, 250 mM ammonium sulfate, 300 mM sucrose, 3 mM MgCl_2_, and 1 mM EGTA] on ice for 5 minutes, digestion buffer [210 mM PIPES pH 6.8, 300 mM sucrose, 50 mM NaCl, 3 mM MgCl_2_, 1 mM EGTA, 2 μl/ml DNase and 125 μl/ml RNase I] at 20°C for 20 minutes, and 2 M NaCl in PBS on ice for 5 minutes. Finally, cells were fixed with 4% paraformaldehyde in PBS and subjected to immunostaining.

### DNA constructs, recombinant proteins and fusion protein purification

To generate recombinant N-terminal His and S dual-tag fused APC fragment M2, the corresponding cDNA for APC (amino acid 1000-1326) was amplified using PCR and subcloned into a pET-30a(+) vector. The DNA construct encoding GST-fused M2-APC (amino acid 959-1338) was a generous gift from Kozo Kaibuchi. Both His-S-M2-APC and GST-M2-APC fusion proteins were expressed and purified as described [51,56]. Expression constructs for GFP fused with full length APC, APC^1417 ^or NLS were generated by PCR amplification of the corresponding regions and insertion into pGFP-C1 vector (Clonetch).

### Affinity-purification of anti-APC-M2 antibody

Anti-APC-M2 rabbit serum was generated by injecting purified S-tag fused M2-APC as the antigen. Serum was acquired and applied to a column of GST-fused M2-APC covalently linked to NHS-Sepharose FastFlow (GE healthcare) according to manufacturer's protocol. After washing with Phosphate Buffered Saline (PBS) plus 0.1% Tween-20 (PBS-T) to remove non-specific proteins, bound antibodies were eluted from the antigen column with 0.2 M Glycine (pH 2.0). Eluted antibodies were dialyzed with PBS containing 10% Glycerol and concentrated to 1 mg/ml using a centrifugal concentrator. Affinity-purified antibody was stored at 4°C with preservatives and is referred to as APC-M2 pAb.

## List of abbreviations

APC: Adenomatous Polyposis Coli; IF: intermediate filament; LC-MS/MS: Liquid Chromatography Tandem Mass Spectrometry; pAb: polyclonal antibody

## Authors' contributions

YW performed all immunoblots, immunoprecipitations, and tissue immunofluorescence staining, and drafted the manuscript. YA purified immunogen S-tag fused M2-APC protein and APC-M2 sera and participated in drafting the Methods section. DBF performed LC-MS/MS analysis and the database search for APC-co-precipitated proteins and contributed to writing the Methods section. RJC participated in manuscript revision and assisted in collection of mouse and human tissues. KLN managed the research, cloned the S-tag fused M2-APC expression construct, carried out the sequential extraction experiment, the shRNA experiment and the immunofluorescence colocalization analysis, drafted the manuscript, and serves as corresponding author. All authors have read and approved the final manuscript.

## Supplementary Material

Additional file 1 **Generation of recombinant M2-APC immunogen**. (A) Schematic diagram of the N-terminal His and S dual-tag fused APC fragment (amino acid 1000-1326) which contains the three 15 amino acid repeats and one 20 amino acid repeat. (B) Purified recombinant M2-APC protein (~50 kDa) used for immunization was resolved by SDS-PAGE and detected using colloidal blue. Click here for file

Additional file 2 **APC-M2 pAb co-precipitates APC binding proteins**. Proteins co-precipitated from HCT116βw cell lysates using APC-M2 pAb were resolved on a 4-12% NUPAGE gel followed by colloidal blue staining. Stars mark the 9 protein bands that were precipitated using the APC-M2 pAb and not using preimmune sera. Click here for file

Additional file 3 **Full list of proteins associated with APC as identified with LC-MS/MS**. Table. Click here for file

Additional file 4 **Negative controls for immunofluorescent microscopy analysis of HCT116βw cells**. (A) Cells processed for conventional immunofluorescent microscopy using either APC-M2 pAb or no primary antibody, followed by goat-anti-mouse Alexa 488 secondary antibody reveal no recognition of the purified rabbit sera by the goat-anti-mouse secondary antibody. (B) Cells processed for conventional immunofluorescent microscopy using mouse monoclonal antibody against lamin B1, pan-keratin, or APC (ali12-28) or no primary antibody, followed by goat-anti-rabbit Alexa 568 secondary antibody reveal no recognition of the mouse monoclonal antibodies by the goat-anti-rabbit secondary antibody. (C) Cells processed for confocal microscopy were stained with goat-anti-rabbit Alexa 568 and goat-anti-mouse Alexa 488 secondary antibodies without primary antibody application. These images served as the negative control for the correlation coefficient analysis. Scale bar, 5 μm. Click here for file
