# Supplementary figures and images for: Novel association of APC with intermediate filaments identified using a new versatile APC antibody
Source: BMC Cell Biol. 2009 Oct 21;10:75. doi: 10.1186/1471-2121-10-75 (PMC2774295; doi:10.1186/1471-2121-10-75)

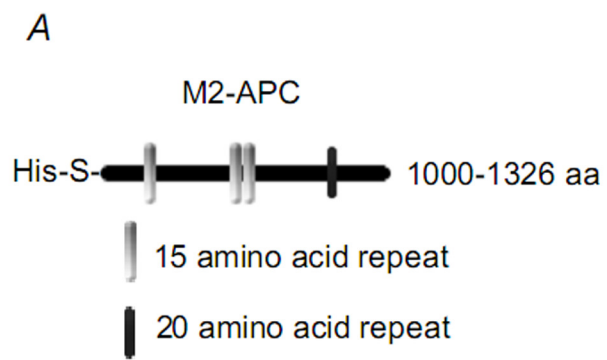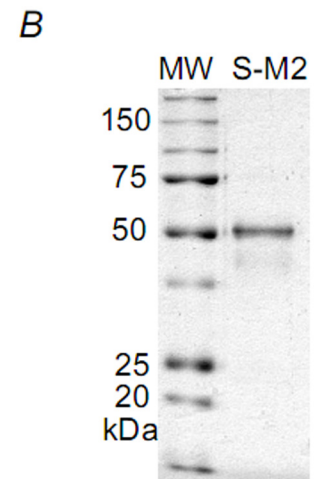

Supplement: Additional file 1 — Generation of recombinant M2-APC immunogen. (A) Schematic diagram of the N-terminal His and S dual-tag fused APC fragment (amino acid 1000-1326) which contains the three 15 amino acid repeats and one 20 amino acid repeat. (B) Purified recombinant M2-APC protein (~50 kDa) used for immunization was resolved by SDS-PAGE and detected using colloidal blue. [file 1471-2121-10-75-S1.pdf]

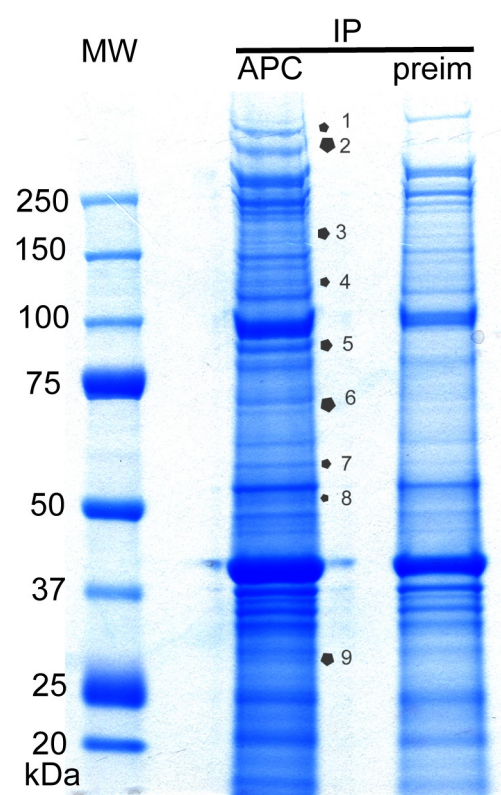

Supplement: Additional file 2 — APC-M2 pAb co-precipitates APC binding proteins. Proteins co-precipitated from HCT116βw cell lysates using APC-M2 pAb were resolved on a 4-12% NUPAGE gel followed by colloidal blue staining. Stars mark the 9 protein bands that were precipitated using the APC-M2 pAb and not using preimmune sera. [file 1471-2121-10-75-S2.pdf]

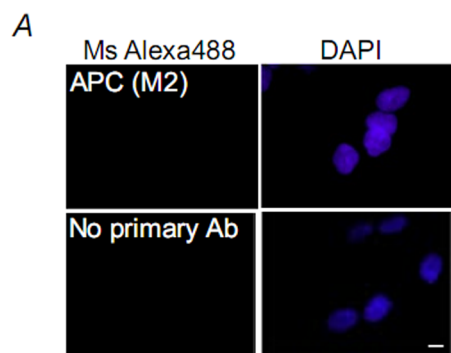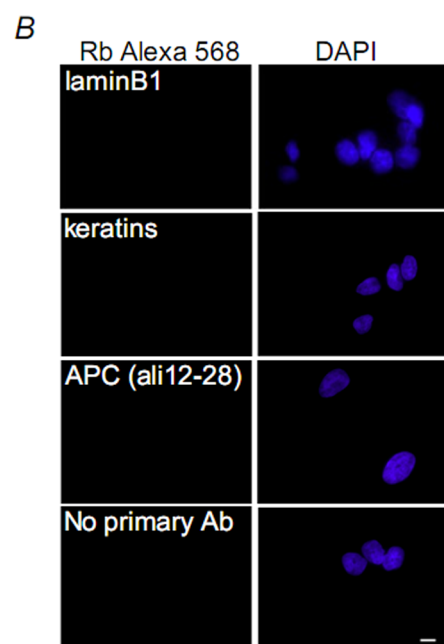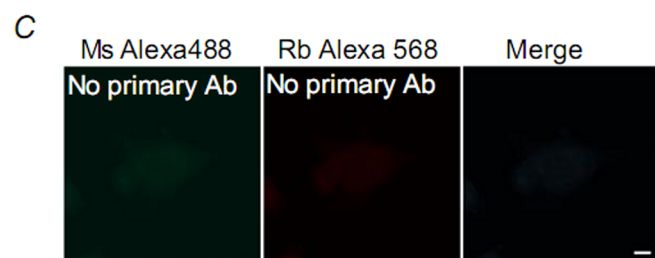

Supplement: Additional file 4 — Negative controls for immunofluorescent microscopy analysis of HCT116βw cells. (A) Cells processed for conventional immunofluorescent microscopy using either APC-M2 pAb or no primary antibody, followed by goat-anti-mouse Alexa 488 secondary antibody reveal no recognition of the purified rabbit sera by the goat-anti-mouse secondary antibody. (B) Cells processed for conventional immunofluorescent microscopy using mouse monoclonal antibody against lamin B1, pan-keratin, or APC (ali12-28) or no primary antibody, followed by goat-anti-rabbit Alexa 568 secondary antibody reveal no recognition of the mouse monoclonal antibodies by the goat-anti-rabbit secondary antibody. (C) Cells processed for confocal microscopy were stained with goat-anti-rabbit Alexa 568 and goat-anti-mouse Alexa 488 secondary antibodies without primary antibody application. These images served as the negative control for the correlation coefficient analysis. Scale bar, 5 μm. [file 1471-2121-10-75-S4.pdf]
