# Supplementary material for: Novel association of APC with intermediate filaments identified using a new versatile APC antibody
Source: BMC Cell Biol. 2009 Oct 21;10:75. doi: 10.1186/1471-2121-10-75 (PMC2774295; doi:10.1186/1471-2121-10-75)
Supplement: Additional file 3 — Full list of proteins associated with APC as identified with LC-MS/MS. Table. [file 1471-2121-10-75-S3.pdf]

Full list of proteins associated with APC as identified with LC-MS/MS

Protein	Accession No.	Peptides found 1st / 2nd run	Functional category
Adenomatous polyposis coli	P25054	89/78	N/A
Lamin B1	Q6DC98	7/6	Intermediate filament
Lamin B2	Q03252	2/3	
Keratin 81	Q14533	5/5	
Keratin 82	Q9NSB4	2/2	
α-actinin-4	O43707	4/3	Cytoskeletal regulation
WD repeat protein 1	O75083	3/3	
Epiplakin	P58107	3/2	
Protein phosphatase 1 regulatory subunit 12A	O14974	3/2	
Tubulin α-ubiquitous chain	P68363	3/2	
Tubulin β chain	P07437	3/2	
Tropomyosin-3	P06753	2/2	
Neurabin-2	Q96SB3	2/2	
α-actinin-1	P12814	2/1	
β-catenin	P35222	70/67	Transcription
RuvB-like 2	Q9Y230	7/8	
RuvB-like 1	Q9Y265	6/6	
RNA-binding protein 14	Q96PK6	6/7	
Ret finger protein isoform β variant	Q59EC6	4/2	

Heterogeneous nuclear ribonucleoprotein K	P61978	3/5	
Pescadillo homolog 1	O00541	2/2	
Protein TFG	Q92734	8/6	Chromosome rearrangement
Ladinin 1	O00515	8/8	Extracellular matrix
40S ribosomal protein S4, Y isoform 1	P22090	8/5	
ATP-dependent RNA helicase DHX15	O43143	7/9	
Splicing factor, arginine/serine-rich 1	Q07955	6/4	
Heterogeneous nuclear ribonucleoprotein M	P52272	4/3	
ATP-dependent RNA helicase DDX3X	O00571	4/2	
Nucleolar RNA helicase 2	Q9NR30	3/2	RNA processing & translation
Splicing factor 3b, subunit 3	Q6NTI8	2/3	
Heterogeneous nuclear ribonucleoprotein Q	O60506	2/2	
Nuclear fragile X mental retardation-interacting protein 2	Q7Z417	3/3	
Transducin beta-like 3 variant	Q59GD6	2/2	
Heat shock 70 kDa protein 2	P54652	7/6	Chaperones
Heat shock 70kDa protein 5	P11021	4/2	
Liprin-β1	Q86W92	3/5	Tumorigenesis
LRCH3 precursor	Q96II8	5/4	Others
Transketolase	P29401	3/3	

(43 proteins; each peptide of which passed Xcorr cutoffs of 2 [+2 ion], 2.5 [+3 ion]; for all proteins at least 2 peptides found each duplicate run)
